# Global burden of vaccine-associated Guillain-Barré syndrome over 170 countries from 1967 to 2023

**DOI:** 10.1038/s41598-024-74729-2

**Published:** 2024-10-19

**Authors:** Yi Deun Jeong, Seoyoung Park, Sooji Lee, Woojin Jang, Jaeyu Park, Kyeongmin Lee, Jinseok Lee, Jiseung Kang, Raphael Udeh, Masoud Rahmati, Seung Geun Yeo, Lee Smith, Hayeon Lee, Dong Keon Yon

**Affiliations:** 1 Department of Medicine, Kyung Hee University College of Medicine, Seoul, South Korea; 2 Center for Digital Health, Medical Science Research Institute, Kyung Hee University Medical Center, Kyung Hee University College of Medicine, 23 Kyungheedae-ro, Dongdaemun-gu, Seoul, 02447 Republic of Korea; 3 Department of Regulatory Science, Kyung Hee University, Seoul, South Korea; 4 Department of Biomedical Engineering, Kyung Hee University, Yongin, South Korea; 5 Division of Sleep Medicine, Harvard Medical School, Boston, MA USA; 6 Department of Anesthesia, Critical Care and Pain Medicine, Massachusetts General Hospital, Boston, MA USA; 7 School of Life Sciences, Faculty of Science, University of Technology Sydney, Ultimo, Australia; 8 Health Service Research and Quality of Life Center (CEReSS), Aix-Marseille Université, Marseille, France; 9 Department of Physical Education and Sport Sciences, Faculty of Literature and Human Sciences, Lorestan University, Khoramabad, Iran; 10 Department of Physical Education and Sport Sciences, Faculty of Literature and Humanities, Vali-E-Asr University of Rafsanjan, Rafsanjan, Iran; 11 Department of Otolaryngology-Head & Neck Surgery, Kyung Hee University Medical Center, Kyung Hee University College of Medicine, Seoul, South Korea; 12 Centre for Health, Performance and Wellbeing, Anglia Ruskin University, East Rd, Cambridge, CB1 1PT UK; 13 Department of Pediatrics, Kyung Hee University Medical Center, Kyung Hee University College of Medicine, Seoul, South Korea

**Keywords:** Global, Guillain-Barré syndrome, Vaccine, Vaccine-associated Guillain-Barré syndrome, World Health Organization, Neurology, Epidemiology

## Abstract

Research on Guillain-Barré syndrome (GBS) as a neurological adverse effect of vaccines on a global scale is scarce, highlighting the need for further investigation to evaluate its long-term impact and associated risk factors comprehensively. Hence, this study aims to assess the global burden of vaccine-associated GBS and its associated vaccines. This study utilized data from VigiBase, the World Health Organization global database of adverse event reports of medicines and vaccines, encompassing the period from 1967 to 2023 (total reports, *n* = 131,255,418) to investigate vaccine-associated GBS. Reported odds ratios (ROR) and information components (IC) were analyzed to assess the association between 19 vaccines and the occurrence of vaccine-associated GBS over 170 countries. We identified 15,377 (8072 males [52.49%]) reports of vaccine-associated GBS among 22,616 reports of all drugs-cause GBS from 1978 to 2023. Cumulative reports of vaccine-associated GBS have been increasing steadily over time, with a notable surge observed since the commencement of COVID-19 vaccines administration in 2020. Most vaccines showed significant associations with GBS such as Ad5-vectored COVID-19 vaccines (ROR, 14.88; IC, 3.66), COVID-19 mRNA vaccines (ROR, 9.66; IC, 2.84), and inactivated whole-virus COVID-19 vaccines (ROR, 3,29; IC 1.69). Influenza vaccines showed the highest association (ROR, 77.91; IC 5.98). Regarding age-and sex-specific risks, the association remained similar regardless of sex, with an increased association observed with advancing age. The mean time to onset was 5.5 days. Amid the COVID-19 pandemic, the reports of GBS surged in response to widespread COVID-19 vaccination. Nonetheless, COVID-19 vaccines exhibited the lowest association compared to other vaccines. Vigilance for at least one-week post-vaccination is crucial, particularly for older adults. Further research is warranted to elucidate the underlying mechanisms linking vaccines and GBS.

**Supplementary Information:**

The online version contains supplementary material available at 10.1038/s41598-024-74729-2.

## Introduction

Vaccines have long been instrumental in dramatically reducing the incidence and mortality rates of infectious diseases^1^. During the COVID-19 pandemic, global vaccination efforts were rapidly implemented^2^. The first vaccines were developed by March 2020 and approved by December 2020^3^, accompanied by ongoing concerns and reports of adverse effects^4^. Guillain Barré syndrome (GBS) has been identified as one of the serious neurological complication after vaccination^5^. GBS, an autoimmune disease associated with significant morbidity, has garnered considerable attention due to a surge in reports during the COVID-19 pandemic^6^. Efforts to address vaccine hesitancy have prompted research into the potential association between COVID-19 vaccines and GBS. However, scarcity of data^7,8^, along with conflicting findings across studies have impeded the development of sufficient consensus.

Before the COVID-19 pandemic, vaccine-associated GBS primarily linked to influenza vaccines^2,9^. Other vaccines, aside from influenza vaccines, that were subjects of debate included those for meningococcus, measles/mumps/rubella (MMR), and human papillomavirus^10^. Nevertheless, the reported cases of GBS associated with these vaccines have been significantly limited, and speculative nature of association persists, contributing to ongoing controversy^10^. Furthermore, recent findings suggest a potential association between the varicella zoster vaccine and GBS^11^, highlighting the ongoing need for comprehensive research into the overall association between vaccines and GBS.

In the absence of a comprehensive global-scale and long-term trend study, our research expands beyond COVID-19 and influenza vaccines to investigate the association between vaccines and GBS, utilizing data from World Health Organization (WHO). This study integrates diverse data, including demographical and epidemiological details, concerning the correlation between vaccines and GBS to elucidate underlying mechanisms. By analyzing comprehensive data, our study aims to enhance the understanding of vaccine-associated GBS, thereby contributing to the development of safer vaccination protocols.

## Methods

### Database

In this study, VigiBase was utilized, the WHO global database of individual case safety reports (ICSRs) developed by the Uppsala Monitoring Center (UMC; WHO Collaborating Center, Uppsala, Sweden)^12–15^. This database encompasses more than 170 countries with 131,255,418 ICSRs of potential side effects of drugs from 1967 to 2023. VigiBase is associated with medical and drug classifications, including terminologies like the Medical Dictionary for Regulatory Activities (MedDRA) for coding adverse events and WHODrug for coding medicines and vaccines. These classifications are effective and accurate analyses of ICSRs of suspected adverse drug reactions (ADRs). This study received approval from the Institutional Review Board at Kyung Hee University and the Uppsala Monitoring Centre (WHO Collaborating Centre) and involved the utilization of de-identified patient data. Informed consent was waived by the Institutional Review Board at Kyung Hee University and the Uppsala Monitoring Centre, as VigiBase does not contain personal information. The dataset is available from the Uppsala Monitoring Centre or WHO through a data use agreement. This research adhered to the ethical guidelines established by relevant national, and institutional review boards for human research and followed the 1975 Helsinki Declaration, as amended in 2008.

### Selection of cases

Vaccine-associated GBS reports documented in VigiBase between 1967 and 2023 were extracted, and the vaccines were categorized into 19 groups: (1) rabies vaccines; (2) yellow fever vaccines; (3) diphtheria, tetanus toxoids, pertussis, polio, and *Hemophilus influenza* type b (DTaP-IPV-Hib) vaccines; (4) pneumococcal vaccines; (5) meningococcal vaccines; (6) pneumococcal vaccines; (7) tuberculosis vaccines; (8) typhoid vaccines; (9) encephalitis vaccines; (10) hepatitis A (HAV) vaccines; (11) hepatitis B (HBV) vaccines; (12) MMR vaccines; (13) rotavirus diarrhea vaccines; (14) varicella zoster vaccines; (15) papillomavirus vaccines; (16) COVID-19 mRNA vaccines; (17) Ad5-vectored COVID-19 vaccines; (18) Inactivated whole-virus COVID-19 vaccines; (19) others (dengue virus, Ebola, leptospira, respiratory syncytial virus, and smallpox vaccines). In our analysis, the total number of drug reports associated with GBS as ADR was 22,616, of which 15,377 reports pertained to the vaccines we are targeting (Table S1). Rather than specifying individual drugs for vaccines associated with GBS, our study utilized medications classified under the anatomical therapeutic chemical (ATC) classification system as designated by WHO (Table S2). Specifically, we included reports pertaining to drugs assigned ATC codes beginning with ‘J07’, which denotes ‘vaccines’.

ADRs are categorized following the MedDRA 26.0 framework in Vigibase, which organizes information into five classes: Lowest Level Terms, Preferred Terms, High-Level Terms, High-Level Group Terms, and System Organ Classes^16–18^. We classified the adverse events associated with GBS from vaccinators into these five distinct classes based on the MedDRA classification system (Table S3). Additionally, we conducted a detailed analysis of the concomitant adverse effects associated with GBS for each vaccine (Table S4).

### Data collection

In our study, we systematically documented instances of presumed vaccine-associated GBS. In the analysis of ICSRs, the covariates of interest include demographic details of patients (i.e., age [0–11, 12–17, 18–44, 45–64, and ≥ 65 years] and sex [male and female]), reporting regions [African, America, South-East Asia, Europe, Eastern Mediterranean, and Western Pacific]), ADRs information (i.e., reporting years [1967–1979, 1980–1989, 1990–1999, 2000–2009, 2010–2019, and 2020–2023], time to onset [TTO] of reaction, outcomes [mild, moderate to severe, and unknown], fatal outcomes of age, reporter qualification [health professional, non-health professional, and unknown], and vaccine information (i.e., vaccines class and basis))^16^.

TTO refers to the interval between the administration of the drug (drug start date) and the onset of the reaction or event (reaction start date). We utilized the TTO calculations following the guidelines provided for VigiBase extraction. All spontaneous reports included at least one suspected vaccine linked to the occurrence of adverse effects following vaccination. The outcome of each event was classified as “mild” or “moderate to severe”. Additionally, case reports originating from physicians, pharmacists, other healthcare professionals, lawyers, other non-healthcare professionals, and consumers (patients) are specified in the ICSRs. The reporter qualification is distinguished as “health professional” or “non-health professional”. To strengthen our analysis, we conducted a subgroup analysis considering cases involving healthcare professionals to examine the association between ADRs and the vaccines.

### Statistical analysis

VigiBase facilitates more robust and rigorous analyses compared to isolated case reports or case series, enabling quantitative comparisons such as disproportionality analysis (case–non-case) to identify which vaccine was significantly associated with GBS. We used two indicators of information component (IC) and reporting odds ratio (ROR)^19,20^, commonly used measures in pharmacovigilance for signaling the disproportionate association between a drug and reported adverse reports^21–23^. It is recommended that drug-adverse event surveillance efforts utilize multiple disproportionality analysis methods, rather than relying on a single approach, to inform decision-making (Table S5)^24^.

The IC was computed assuming a Bayesian analysis for case-non-case analysis^21^. It serves as an indicator value for disproportionate reporting, comparing observed and expected ADR associations to identify the drug-ADR signals with a probability difference from the background data^25^. ROR was calculated using the following formula: ROR = (a/b)/(c/d), where “a” represents the number of reports for a certain adverse drug reaction, “b” is the number of reports for all other ADRs with a specific drug, “c” is the number of all reports for certain ADRs not related to a specific drug, and “d” is the number of all reports not related to both specific ADRs and drugs. An IC_025,_ the value representing the lower end of the 95% confidence interval of the IC, greater than 0.00 and ROR > 1.00 indicate statistical significance^25^. It means that cases are more reported with the drug of interest than with other drugs, same as the greater the disproportionality. The IC_025_ and ROR, being statistical estimates, should always be presented and interpreted with a 95% confidence interval (95% CI). All analyses were performed utilizing SAS software (version 9.4; SAS Inc., Cary, NC, USA)^26,27^.

## Results

### Overall analysis

**Fig. 1:**
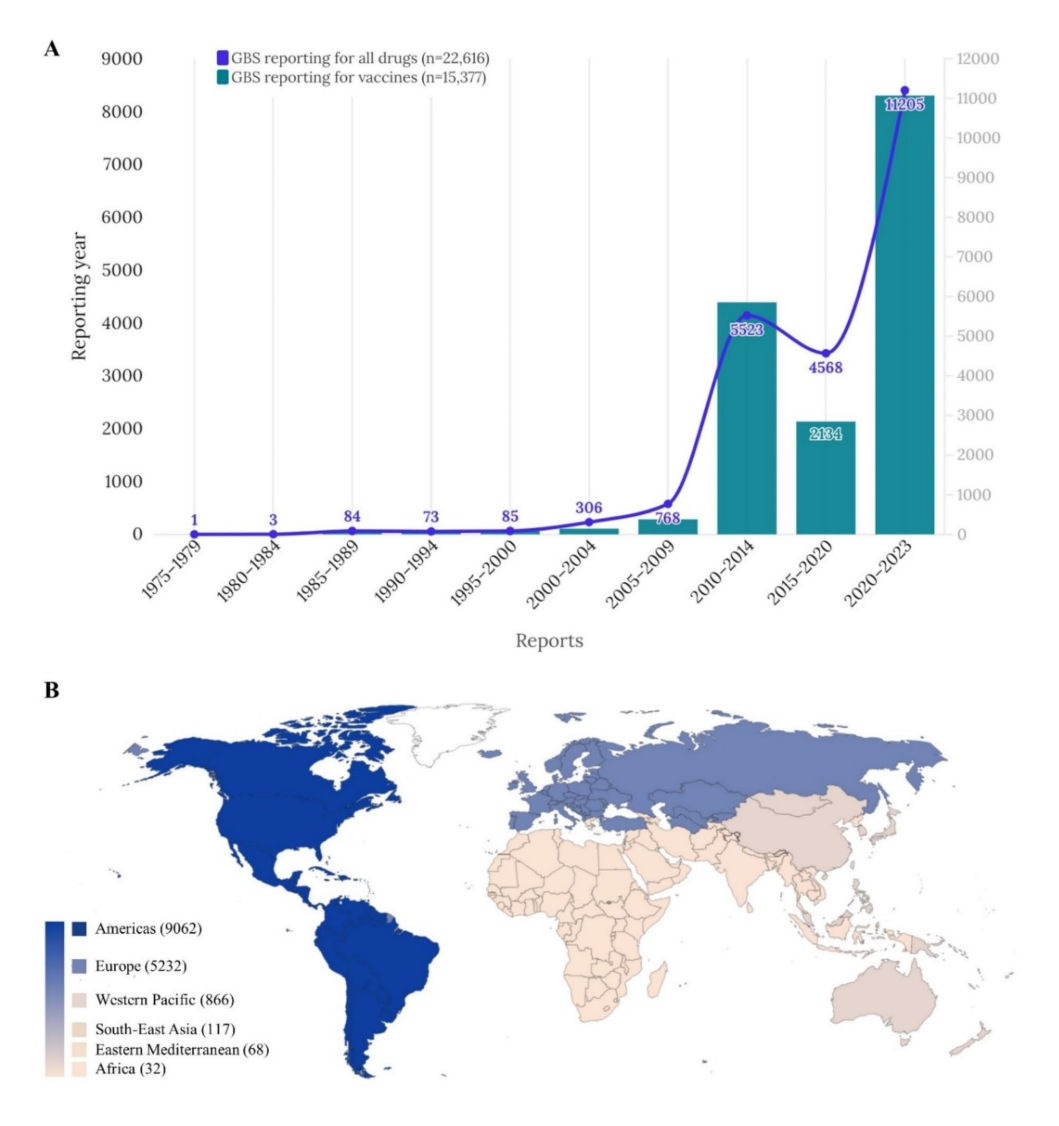
Temporal trends (**A**) and global distribution (**B**) of vaccine-associated GBS adverse events by continent (total *n* = 15,377). *GBS* Guillain-Barré syndrome.

Among the 8,010,602 reports in the full database, a disproportionality analysis was conducted on a dataset comprising 15,377 cases (8072 male [52.49%]) of vaccine-associated GBS documented in VigiBase between 1978 and 2023 (Table 1). We categorized the reported incidents into six geographical regions, as shown in Fig. 1. Reports from the region of the Americas constituted over half of the total (58.93%), followed by European region (34.02%), and Western Pacific region (5.63%). The majority of reports were associated with COVID-19 mRNA vaccines (29.17%), followed by influenza vaccines (26.25%), and Ad5-vectored COVID-19 vaccines (16.23%). The reports of GBS were distributed across the age groups of 0–11 years (6.43%), 12–17 years (5.22%), 18–44 years (23.26%), 45–64 years (28.74%), and 65 years and older (23.68%). Mean TTO was 5.47 days and standard deviation 41.72 (Table 1). A sub-analysis of vaccine-associated GBS, based exclusively on reports from healthcare professionals, is presented in Table 2. TTO of individual vaccines are shown in Table 3.

**Table 1:** Baseline characteristics of reports on vaccine-associated GBS adverse event, in the VigiBase, a WHO pharmacovigilance database between 1967 and 2023 (*n* = 15,377).

Variables	Number (%)
Region reporting	
African region	32 (0.21)
Region of the Americas	9062 (58.93)
South-East Asia region	117 (0.76)
European region	5232 (34.02)
Eastern Mediterranean region	68 (0.44)
Western Pacific region	866 (5.63)
Reporting year	
1967–1979	1 (0.01)
1980–1989	48 (0.31)
1990–1999	107 (0.70)
2000–2009	392 (2.55)
2010–2019	6525 (42.43)
2020–2023	8304 (54.00)
Reporter qualification	
Health professional	4833 (31.43)
Non-health professional	1952 (12.69)
Unknown	8592 (55.88)
Sex	
Male	8072 (52.49)
Female	7097 (46.15)
Unknown	208 (1.46)
Age, years	
0–11	989 (6.43)
12–17	803 (5.22)
18–44	3577 (23.26)
45–64	4419 (28.74)
≥ 65	3641 (23.68)
Unknown	1948 (12.67)
TTO, days	
Mean (SD)	5.47 (41.72)
Vaccine class	
Routine	7974 (51.86)
DTaP-IPV-Hib vaccines	1238 (8.05)
Meningococcal vaccines	355 (2.31)
Pneumococcal vaccines	485 (3.15)
Tuberculosis vaccines	10 (0.07)
Encephalitis vaccines	143 (0.93)
Influenza vaccines	4037 (26.25)
Hepatitis A vaccines	350 (2.28)
Hepatitis B vaccines	300 (1.95)
MMR vaccines	271 (1.76)
Rotavirus diarrhea vaccines	15 (0.10)
Varicella zoster vaccines	360 (2.34)
Papillomavirus vaccines vaccines	410 (2.67)
Non-routine	7378 (47.98)
Rabies vaccines	71 (0.46)
Yellow fever vaccines	119 (0.77)
Typhoid vaccines	123 (0.80)
COVID-19 mRNA vaccines vaccines	4486 (29.17)
Ad5-vectored COVID-19 vaccines	2496 (16.23)
Inactivated whole-virus COVID-19 vaccines	83 (0.54)
Others*	25 (0.16)
Fatal outcomes	
Mild	6906 (44.91)
Moderate to severe	118 (0.77)
Unknown	8353 (54.32)
Fatal, age, years	
0–11	2 (1.69)
12–17	0 (0.00)
18–44	7 (5.93)
45–64	16 (13.56)
≥ 65	87 (74.58)
Unknown	5 (4.24)
Basis	
Single drug suspected	15,363 (99.91)
Interacting	14 (0.09)

*DTaP-IPV-Hib* diphtheria, tetanus toxoids, pertussis, polio, and *Hemophilus influenza* type b, *GBS* Guillain-Barré syndrome, *MMR* measles, mumps, and rubella, *TTO* time to onset, *WHO* World Health Organization.

*Others: dengue virus, Ebola, leptospira, respiratory syncytial virus, and smallpox vaccines.

**Table 2:** Sub-analysis of the disproportionate occurrence in vaccine-related GBS adverse events disproportionality.

Vaccine-associated GBS				
	Total cases (*n* = 15,377)		Case reported from health professional report (*n* = 4833)	
	ROR (95% CI)	IC (IC_025_)	ROR (95% CI)	IC (IC_025_)
Total	**32.72 (31.82 to 33.65)**	**3.48 (3.45)**	0.82 (0.80 to 0.85)	− 0.22 (− 0.27)
Sex difference				
Male	**31.68 (30.48 to 32.93)**	**3.43 (3.39)**	0.83 (0.79 to 0.86)	− 0.21 (− 0.28)
Female	**33.92 (32.51 to 35.39)**	**3.45 (3.41)**	0.73 (0.70 to 0.77)	− 0.35 (− 0.42)
Vaccine types				
DTaP-IPV-Hib vaccines	**9.31 (8.80 to 9.86)**	**3.14 (3.05)**	0.75 (0.69 to 0.83)	− 0.40 (− 0.56)
Meningococcal vaccines	**13.90 (12.51 to 15.43)**	**3.75 (3.57)**	0.60 (0.47 to 0.77)	− 0.73 (− 1.15)
Pneumococcal vaccines	**10.47 (9.57 to 11.46)**	**3.34 (3.19)**	**0.70 (0.59 to 0.83)**	− 0.51 (− 0.80)
Tuberculosis vaccines	1.69 (0.91 to 3.14)	0.71 (− 0.37)	0.17 (0.06 to 0.45)	− 2.43 (− 4.20)
Encephalitis vaccines	**2.26 (1.91 to 2.66)**	**1.16 (0.89)**	**8.20 (6.84 to 9.83)**	**2.98 (2.67)**
Influenza vaccines	**77.91 (75.30 to 80.62)**	**5.98 (5.93)**	**5.08 (4.80 to 5.38)**	**2.27 (2.18)**
Hepatitis A vaccines	**32.67 (29.39 to 36.32)**	**4.94 (4.76)**	**1.93 (1.56 to 2.39)**	**0.94 (0.58)**
Hepatitis B vaccines	**15.99 (14.27 to 17.92)**	**3.94 (3.75)**	1.07 (0.86 to 1.33)	0.10 (− 0.27)
MMR vaccines	**7.03 (6.24 to 7.92)**	**2.78 (2.58)**	0.48 (0.38 to 0.60)	− 1.04 (− 1.42)
Rotavirus diarrhea vaccines	1.03 (0.62 to 1.72)	0.05 (− 0.82)	0.10 (0.05 to 0.22)	− 3.20 (− 4.62)
Varicella zoster vaccines	**9.62 (8.67 to 10.68)**	**3.23 (3.05)**	0.46 (0.37 to 0.58)	− 1.10 (− 1.49)
Papillomavirus vaccines vaccines	**17.60 (15.96 to 19.41)**	**4.08 (3.92)**	**4.16 (3.77 to 4.58)**	**2.03 (1.86)**
Rabies vaccines	**28.24 (22.36 to 35.67)**	**4.56 (4.17)**	**3.54 (2.55 to 4.90)**	**1.77 (1.21)**
Yellow fever vaccines	**24.67 (20.60 to 29.56)**	**4.48 (4.17)**	**2.34 (1.76 to 3.13)**	**1.21 (0.72)**
Typhoid vaccines	**42.52 (35.59 to 50.80)**	**5.17 (4.87)**	**2.56 (1.79 to 3.67)**	**1.32 (0.71)**
COVID-19 mRNA vaccines vaccines	**9.66 (9.33 to 10.00)**	**2.84 (2.80)**	0.32 (0.30 to 0.34)	− 1.45 (− 1.56)
Ad5-vectored COVID-19 vaccines	**14.88 (14.26 to 15.53)**	**3.66 (3.59)**	**1.38 (1.30 to 1.46)**	**0.43 (0.33)**
Inactivated whole-virus COVID-19 vaccines	**3.29 (2.65 to 4.09)**	**1.69 (1.33)**	0.60 (0.47 to 0.76)	− 0.73 (− 1.13)

*DTaP-IPV-Hib* diphtheria, tetanus toxoids, pertussis, polio, and *Hemophilus influenza* type b, *GBS* Guillain-Barré syndrome, *IC* information component, *MMR* measles, mumps, and rubella, *ROR* reported odds ratio.

Bold style indicates when the value of IC_025_ is greater than 0.00 or the lower end of the ROR 95% CI is greater than 1.00. This means it is statistically significant.

**Table 3:**
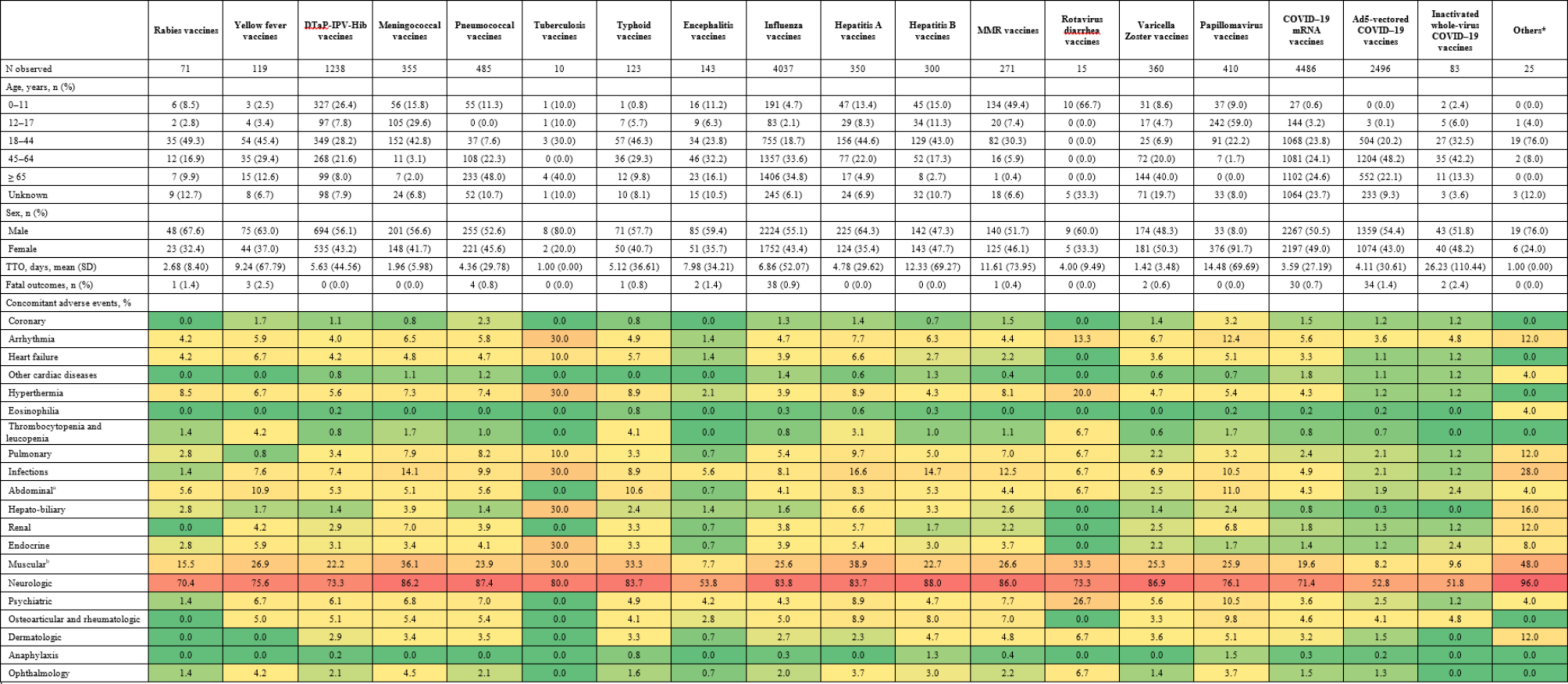
Detail reports for each vaccine associated with GBS and concomitant adverse events (heatmap).

*DTaP-IPV-Hib* diphtheria, tetanus toxoids, pertussis, polio, and *Hemophilus influenza* type b, *GBS* Guillain-Barré syndrome, *MMR* measles, mumps, and rubella, *TTO* time to onset.

### **Disproportionality analysis of vaccine-associated GBS**

Most vaccines, except rotavirus and tuberculosis vaccines, showed a significant association with GBS (Table 4). Influenza vaccines had the highest association with GBS (ROR, 77.91 [95% CI, 75.30–80.62]; IC, 5.98 [IC_025,_ 5.93]), followed by typhoid vaccines (ROR, 42.52 [95% CI, 35.59–50.80]; IC, 5.17 [IC_025,_ 4.87]), hepatitis A vaccines (ROR, 32.67 [95% CI, 29.39–36.32]; IC, 4.94 [IC_025,_ 4.76]), rabies vaccines (ROR, 28.24 [95% CI, 22.36–35.67]; IC, 4.56 [IC_025,_ 4.17]), yellow fever vaccines (ROR, 24.67 [95% CI, 20.6–29.56]; IC, 4.48 [IC_025,_ 4.17]), papillomavirus vaccines (ROR, 17.60 [95% CI, 15.96–19.41]; IC, 4.08 [IC_025,_ 3.92]), hepatitis B vaccines (ROR, 15.99 [95% CI, 14.27–17.92]; IC, 3.94 [IC_025,_ 3.75]), ad5-vectored COVID-19 vaccines (ROR, 14.88 [95% CI, 14.26–15.53]; IC, 3.66 [IC_025,_ 3.59]), meningococcal vaccines (ROR, 13.90 [95% CI, 12.51–15.43]; IC, 3.75 [IC_025,_ 3.57]), pneumococcal vaccines (ROR, 10.47 [95% CI, 9.57–11.46]; IC, 3.34 [IC_025,_ 3.19]), COVID-19 mRNA vaccines (ROR, 9.66 [95% CI, 9.33–10.00]; IC, 2.84 [IC_025,_ 2.80]), varicella zoster vaccines (ROR, 9.62 [95% CI, 8.67–10.68]; IC, 3.23 [IC_025,_ 3.05]), DTaP-IPV-Hib vaccines (ROR, 9.31 [95% CI, 8.8–9.86]; IC, 3.14 [IC_025,_ 3.05]), MMR vaccines (ROR, 7.03 [95% CI, 6.24–7.92]; IC, 2.78 [IC_025,_ 2.58]), inactivated whole-virus COVID-19 vaccines (ROR, 3.29 [95% CI, 2.65–4.09]; IC, 1.69 [IC_025,_ 1.33]), and encephalitis vaccines (ROR, 2.26 [95% CI, 1.91–2.66]; IC, 1.16 [IC_025,_ 0.89]).

**Table 4:** Analysis of the disproportionate occurrence in vaccine-related GBS adverse events disproportionality.

	Total	Vaccine-associated GBS			IC (IC_025_) based on age, years				
		Observed	ROR (95% CI)	IC (IC_025_)	0–11 years	12–17 years	18–44 years	45–64 years	≥ 65 years
Total	8,059,284	15,377	**32.72 (31.82 to 33.65)**	**3.48 (3.45)**	**1.92 (1.81)**	**2.86 (2.75)**	**2.93 (2.87)**	**3.67 (3.62)**	**4.19 (4.13)**
Sex difference									
Male	2,955,730	8072	**31.68 (30.48 to 32.93)**	**3.43 (3.39)**	**1.98 (1.82)**	**2.91 (2.71)**	**2.94 (2.86)**	**3.87 (3.80)**	**4.25 (4.18)**
Female	4,965,554	7097	**33.92 (32.51 to 35.39)**	**3.45 (3.41)**	**1.83 (1.68)**	**2.79 (2.64)**	**2.93 (2.85)**	**3.55 (3.48)**	**4.15 (4.06)**
Vaccine types									
Routine	2,494,608	7974	**28.18 (27.42 to 28.96)**	**4.21 (4.17)**	**1.92 (1.81)**	**3.32 (3.19)**	**5.15 (5.07)**	**5.57 (5.50)**	**5.57 (5.50)**
DTaP-IPV-Hib vaccines	812,141	1238	**9.31 (8.80 to 9.86)**	**3.14 (3.05)**	**1.44 (1.26)**	**3.24 (2.90)**	**5.21 (5.04)**	**5.45 (5.25)**	**5.26 (4.93)**
Meningococcal vaccines	150,715	355	**13.90 (12.51 to 15.43)**	**3.75 (3.57)**	**1.78 (1.33)**	**3.11 (2.79)**	**5.63 (5.36)**	**3.63 (2.61)**	**3.61 (2.31)**
Pneumococcal vaccines	274,186	485	**10.47 (9.57 to 11.46)**	**3.34 (3.19)**	**1.00 (0.55)**	− 1.17 (− 11.49)	**3.76 (3.21)**	**4.36 (4.05)**	**4.29 (4.07)**
Tuberculosis vaccines	34,441	10	**1.69 (0.91 to 3.14)**	0.71 (− 0.37)	− 1.79 (− 5.57)	0.20 (− 3.58)	**2.38 (0.31)**	− 0.29 (− 10.61)	**2.63 (0.86)**
Encephalitis vaccines	20,806	143	**2.26 (1.91 to 2.66)**	**1.16 (0.89)**	**3.52 (2.68)**	**3.38 (2.24)**	**4.43 (3.86)**	**5.31 (4.82)**	**4.93 (4.23)**
Influenza vaccines	368,978	4037	**77.91 (75.30 to 80.62)**	**5.98 (5.93)**	**4.00 (3.76)**	**3.90 (3.53)**	**5.48 (5.36)**	**6.24 (6.15)**	**6.40 (6.31)**
Hepatitis A vaccines	63,173	350	**32.67 (29.39 to 36.32)**	**4.94 (4.76)**	**3.19 (2.71)**	**3.40 (2.78)**	**5.57 (5.31)**	**5.40 (5.02)**	**4.47 (3.65)**
Hepatitis B vaccines	110,384	300	**15.99 (14.27 to 17.92)**	**3.94 (3.75)**	**2.59 (2.10)**	**3.17 (2.60)**	**4.30 (4.01)**	**4.23 (3.77)**	**3.5 (2.28)**
MMR vaccines	226,915	271	**7.03 (6.24 to 7.92)**	**2.78 (2.58)**	**2.06 (1.77)**	**2.45 (1.70)**	**4.28 (3.91)**	**3.65 (2.81)**	1.40 (− 2.38)
Rotavirus diarrhea vaccines	82,471	15	1.03 (0.62 to 1.72)	0.05 (− 0.82)	− 0.29 (− 1.37)	− 0.01 (− 10.34)	− 0.04 (− 10.36)	− 0.02 (− 10.34)	0.00 (− 10.33)
Varicella zoster vaccines	216,070	360	**9.62 (8.67 to 10.68)**	**3.23 (3.05)**	**1.30 (0.70)**	**2.48 (1.66)**	**3.44 (2.77)**	**3.08 (2.69)**	**3.88 (3.60)**
Papillomavirus vaccines	134,328	410	**17.60 (15.96 to 19.41)**	**4.08 (3.92)**	**3.44 (2.90)**	**3.29 (3.07)**	**4.10 (3.75)**	**3.53 (2.23)**	− 0.05 (− 10.37)
Non-routine	5,498,958	7378	**11.08 (10.78 to 11.39)**	**2.96 (2.92)**	**1.91 (1.38)**	**1.82 (1.56)**	**2.06 (1.98)**	**2.98 (2.91)**	**3.41 (3.33)**
Rabies vaccines	14,708	71	**28.24 (22.36 to 35.67)**	**4.56 (4.17)**	**3.15 (1.73)**	1.78 (− 0.81)	**4.32 (3.76)**	**3.66 (2.68)**	**3.63 (2.33)**
Yellow fever vaccines	28,252	119	**24.67 (20.60 to 29.56)**	**4.48 (4.17)**	1.17 (− 0.90)	**2.06 (0.30)**	**4.15 (3.69)**	**4.69 (4.12)**	**4.48 (3.61)**
Typhoid vaccines	17,021	123	**42.52 (35.59 to 50.80)**	**5.17 (4.87)**	1.03 (− 2.76)	**3.33 (2.03)**	**4.78 (4.34)**	**5.12 (4.57)**	**4.32 (3.34)**
COVID-19 mRNA vaccines	4,009,826	4486	**9.66 (9.33 to 10.00)**	**2.84 (2.80)**	**2.01 (1.36)**	**2.30 (2.03)**	**2.07 (1.97)**	**2.51 (2.41)**	**3.28 (3.18)**
Ad5-vectored COVID-19 vaccines	1,266,581	2496	**14.88 (14.26 to 15.53)**	**3.66 (3.59)**	− 0.78 (− 11.10)	1.53 (− 0.54)	**2.49 (2.34)**	**4.01 (3.92)**	**4.15 (4.01)**
Inactivated whole-virus COVID-19 vaccines	162,570	83	**3.29 (2.65 to 4.09)**	**1.69 (1.33)**	1.93 (− 0.66)	**2.78 (1.21)**	**0.87 (0.23)**	**2.08 (1.52)**	**1.45 (0.43)**

*DTaP-IPV-Hib* diphtheria, tetanus toxoids, pertussis, polio, and *Hemophilus influenza* type b, *GBS* Guillain-Barré syndrome, *IC* information component, *MMR* measles, mumps, and rubella, *ROR* reported odds ratio, *TTO* time to onset, *WHO* World Health Organization.

Bold style indicates when the value of IC_025_ is greater than 0.00 or the lower end of the ROR 95% CI is greater than 1.00. This means it is statistically significant.

Upon examining the correlation between GBS and total vaccines across different age groups, a significant association was evident in all age groups. The significance of this correlation was observed to increase with age. The highest association was found in those aged 65 and above (IC, 4.19 [IC_025_, 4.13]), followed by the age group between 45 and 64 years (IC, 3.67 [IC_025_, 3.62]), 18–44 years (IC, 2.93 [IC_025,_ 2.87]), 12–17 years (IC, 2.86 [IC_025,_ 2.75]), and 0–11 years (IC, 1.92 [IC_025,_ 1.81]). Upon analyzing individual vaccines, influenza, varicella zoster, COVID-19 mRNA, and ad5-vectored COVID-19 vaccines exhibited higher association with the older age group. Inactivated whole-virus COVID-19 vaccines exclusively showed the highest association with the age group between 12 and 17 years. The other vaccines exhibited the highest association with the age group between 18 and 64 years. For instance, rabies vaccines (IC, 4.32 [IC_025,_ 3.76]), yellow fever vaccines (IC, 4.69 [IC_025,_ 4.12]), DTaP-IPV-Hib vaccines (IC, 5.45[IC_025,_ 5.25]), meningococcal vaccines (IC, 5.63 [IC_025,_ 5.36]), pneumococcal vaccines (IC, 4.36 [IC_025,_ 4.05]), typhoid vaccines (IC, 5.12 [IC_025,_ 4.57]), encephalitis vaccines (IC, 5.31 [IC_025,_ 4.82]), hepatitis A vaccines (IC, 5.57 [IC_025,_ 5.31]), hepatitis B vaccines (IC, 4.30 [IC_025,_ 4.01]), MMR vaccines (IC, 4.28 [IC_025,_ 3.91]), and papillomavirus vaccines (IC, 4.10 [IC_025,_ 3.75]).

Among routinely administered vaccines, with the exception of papillomavirus vaccines, which are recommended to be administered beginning at ages 11 or 12, most show a tendency for a stronger association with increasing age. For papillomavirus vaccines, the highest level of association is observed in the 18–44 age group. Among non-routine vaccines, COVID-19 vaccines tend to show a stronger association with increasing age, likely due to the administration of booster doses to older age groups. Other non-routine vaccines, such as rabies, yellow fever, typhoid, encephalitis, and tuberculosis vaccines, tend to exhibit the highest association in the 18–64 age group, possibly due to higher societal engagement and travel activities across these age ranges.

Upon examining the differences based on sex, it was observed that there is no significant disparity in the association with GBS between males (ROR, 31.68 [95% CI, 30.48–32.93]; IC, 3.43 [IC_025,_ 3.39]) and females (ROR, 33.92 [95% CI, 32.51–35.39]; IC, 3.45 [IC_025,_ 3.41]). Both males (IC, 4.25 [IC_025_, 4.18]) and females (IC, 4.15 [IC_025_, 4.06]) exhibited the highest association with the age group over 65 years, with a stronger association observed as age increased. Detailed description of reports regarding vaccine-associated GBS is provided in Table 3.

### Cumulative report analysis

**Fig. 2:**
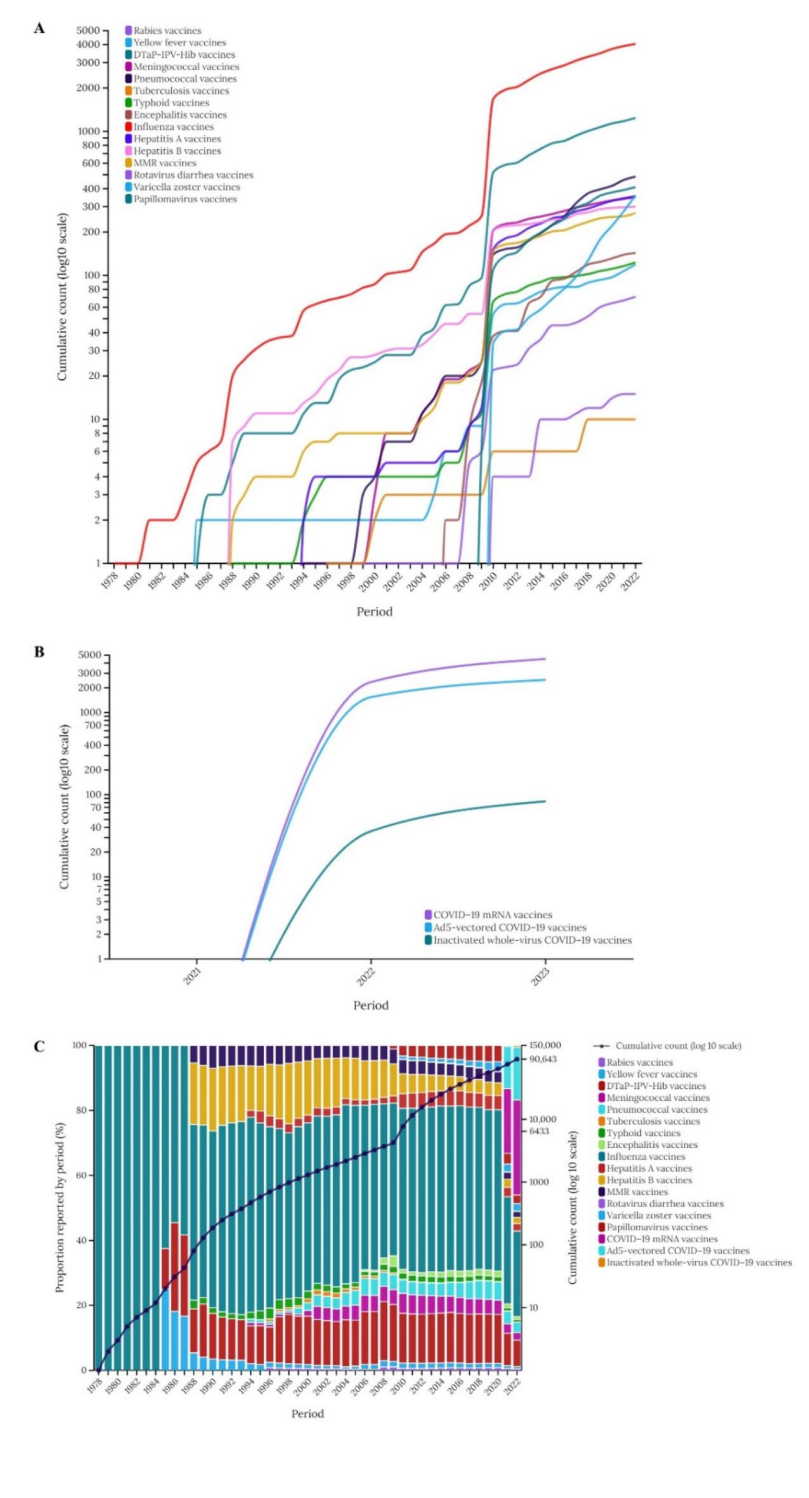
Cumulative number of reports of GBS adverse events per year in association with different vaccines (**A**–**C**). *GBS,*Guillain-Barré syndrome.

The cumulative number of vaccine-associated GBS reports is shown in Fig. 2. Before 2010, only a few reports were documented, but afterwards, the emergence of reports associated with several vaccines led to a dramatic overall increase in reports. Furthermore, from mid-2020, the rapid increase in reports escalated with the introduction of COVID-19 related vaccines, among which the COVID-19 mRNA vaccines accounted for the highest proportion, followed by Ad5-vectored COVID-19 vaccines.

## Discussion

### Key finding

In summary, our study conducted a global investigation into vaccine-associated GBS, utilizing data from the WHO international pharmacovigilance database. During the COVID-19 pandemic, the numbers of reports of GBS associated with COVID-19 vaccines significantly increased. One notable aspect here is that, contrary to the substantial number of reports, the associations between COVID-19 vaccines and GBS were found to be among the lowest. No significant disproportion between males and females was observed overall, unlike conventional GBS epidemiology. However, within the 45–64 age group, a notable sex disproportionality emerged. The increased risk of GBS with advancing age aligns with natural epidemiological patterns, a tendency observed across various individual vaccines such as influenza, varicella zoster, COVID-19 mRNA, and ad5-vectored COVID-19 vaccines. The mean time to onset was 5.47 days, consistent with the finding that GBS typically occurs within two weeks after vaccination.

### Plausible underlying mechanisms

Before the COVID-19 pandemic, three mechanisms contributing to immune system activation were implicated in explaining vaccine-associated GBS^28^. The molecular mimicry hypothesis has garnered significant attention. This theory suggests that epitopes within a vaccine have the potential to elicit the production of antibodies and/or T cells that can cross-react with epitopes present on myelin or axonal glycoproteins^28,29^. Damage to axonal or myelin membranes could potentially occur through direct mediation by the vaccine virus or vaccine-related components^28^, along with genetic predispositions such as human leukocyte antigen polymorphism^28,29^.

However, cases of GBS occurring after COVID-19 vaccination exhibit a distinct pattern compared to other vaccines. Particularly, the Ad5-vectored COVID-19 vaccine has been associated with a higher incidence of GBS^30–33^. Additionally, in patients who developed GBS following Ad5-vectored COVID-19 vaccines, lower levels of antiganglioside antibodies were found, suggesting a classification of GBS into the acute inflammatory demyelinating polyneuropathy (AIDP) subtype^34,35^. Therefore, suspicion has shifted from antiganglioside antibodies, which traditionally played a primary role in the pathogenesis, to molecular mimicry antigens that may share structural similarities with adenoviral vectors^36^. This is because mRNA vaccines have been associated with fewer reports of GBS^29,33^ and have even been suggested to be protective in some studies^37^. However, suspicion towards adenovirus is tempered by the historically low association between adenovirus and GBS^38^. Consequently, this remains a speculative hypothesis. Additionally, abnormal splice variants, contaminated proteins, or other vaccine components may also elicit an immune response in GBS, but the precise antigenic targets necessitate further investigation^29^.

### Clinical and policy implications

When considering individual vaccines, such as influenza, varicella zoster, COVID-19 mRNA, and ad5-vectored COVID-19 vaccines, the association appears to strengthen with advancing age. However, when examining the age-specific associations of other individual vaccines, the highest correlation is observed within the 18–64 age group. Our findings indicate that symptoms of GBS typically manifest within approximately 5.5 days, suggesting onset occurs within a week. While existing literature has highlighted differences in onset times between vaccines, with COVID-19 vaccines generally reported within two weeks^29,34^ and influenza vaccines within 2–4 weeks^10^, our global data indicate an average onset within one week post-vaccination. Therefore, healthcare professionals should consider the possibility that vaccines may be a contributing factor in cases of GBS, particularly in older patients, when there is a history of vaccination within the preceding two weeks in a clinical setting.

In low-income countries, timely treatment options proven effective for GBS, such as immunoglobulin or plasma exchange therapy, may be inaccessible^39^. However, among the known treatments for GBS, these two are recognized as beneficial^40^. Delayed treatment may prolong recovery and impede complete recovery^9,40^. Linear findings suggest a notably high mortality rate, such as 17% in countries with limited resources like Bangladesh^9^. In such nations, while timely treatment with immunotherapy remains crucial, considering the cost-effectiveness, exploring the efficacy of alternative options like exchange transfusion or small volume plasma exchange therapy appears necessary^9,39^. Moreover, in cases of GBS reported following COVID-19 vaccination, the predominant subtype observed is often AIDP^29^. However, in instances of GBS attributed to prior vaccines, notably the 1976 influenza vaccine, a subtype resembling acute motor axonal neuropathy (AMAN) has been hypothesized, particularly with the detection of induced antibodies to ganglioside GM1 antibody^41^. Yet, research examining whether AIDP and AMAN warrant identical treatment remains scarce and uncertain^39^, necessitating further investigation. Additionally, continuous surveillance and research are warranted to ascertain the predominant subtype following the administration of each vaccine.

While our research has identified an association between most vaccines and GBS, it is important to note that studies have consistently shown a higher risk of GBS occurrence during SARS-CoV-2 and influenza infections compared to receiving the vaccines^9,37,42^. Particularly in the case of influenza infection, research indicates a 4–7 folds increase in GBS occurrence, underscoring the benefit of vaccination^2,39^. Moreover, recent studies have suggested that receiving vaccines such as the varicella zoster vaccine can reduce the risk of GBS compared to infection^11^. Given the approximately 1.8% mortality rate documented in previous literature for vaccine-associated GBS^36^, and considering the typical mortality rate of around 5% for GBS cases, along with the absence of any increased risk of GBS relapse following vaccination^9^, vaccination presents a clear overall benefit. However, in high-risk groups, careful observation is warranted, and prompt treatment seems imperative.

### Strengths and limitations

Our study has several limitations. The majority of reports regarding vaccine-associated GBS originate from Europe and America, highlighting the potential for underreporting in low to middle-income countries. Prior to the 2009 influenza pandemic, surveillance systems were insufficiently established, and awareness of reporting practices was limited^43^. As illustrated in Fig. 1A, a notable surge in reporting is evident from 2010 onwards, indicating enhanced awareness and reporting efforts. Despite consensus regarding the association between GBS and the H1N1 influenza vaccine used in 1976^9,40^, the relatively low reporting numbers before 2010 suggest a reporting bias likely influenced by awareness of surveillance systems and reporting to the global system. Additionally, since GBS typically arises following antecedent infections^39^, clinicians encountering patients with GBS might find it challenging to attribute the cause to vaccines.

There exists a possibility that GBS diagnoses were expedited, and differential diagnoses resembling GBS may need to receive more consideration. In particular, cases not precisely meeting the Brighton Collaboration GBS criteria or labeled as probable GBS might have been included, potentially introducing bias^44^. Despite our intention to mitigate reporting bias by encompassing all GBS subtypes to comprehensively assess its impact, this endeavor may have been insufficient. Consequently, vaccines previously perceived as unrelated, such as diphtheria, tetanus toxoids, pertussis and COVID-19 mRNA vaccines^10,37^, could plausibly exhibit associations due to these factors. There are also possibilities that GBS following live attenuated vaccinations are due to the immunization failure, not by the vaccination itself. Moreover, due to the limitations of the self-reporting system in VigiBase, the tendency to report symptoms when they are more severe cannot be underestimated.

Furthermore, our analysis may have failed to accurately incorporate age as a risk factor for GBS. Specifically, vaccines such as the rotavirus vaccine, primarily administered to infants^45^, might have led to a reduced likelihood of GBS occurrence. Most routine vaccines, except for papillomavirus vaccines, are primarily administered between the ages of 0 and 11 years. However, in our analysis, the level of association with GBS tends to increase with age, consistent with the natural epidemiology of GBS, where its incidence rises with age. This underscores that our analysis did not account for age as a confounding factor. Additionally, COVID-19 vaccines show a higher association with increasing age; however, the higher prevalence of booster doses among older age groups may result in differences in the number of doses administered across age groups^46^, which could, in turn, contribute to the stronger association observed in older populations. Moreover, the irregular administration of the tuberculosis vaccine in regions like America and Europe^47^, which constitute the main reporting areas, makes it challenging to evaluate its precise impact on GBS.

Despite these limitations, our study conducted a comprehensive and long-term analysis of the association between vaccines and GBS on a global scale, utilizing data from the WHO pharmacovigilance database. By analyzing the association between vaccines and GBS since 1967, we evaluated the global burden using two disproportionality analysis measures, thereby mitigating the inherent limitations of the spontaneous reporting system. Unlike previous studies, which were constrained by geographical limitations and insufficient data, our research overcame these setbacks by simultaneously analyzing the overall association between all vaccines and GBS. This study, utilizing the WHO database, aimed to provide healthcare practitioners with more accurate data on the adverse effects of vaccines, thereby contributing to the development of more tailored vaccination protocols for patients.

## Conclusion

Our study, utilizing WHO data, observed a notable increase in reports of vaccine-associated GBS during the COVID-19 pandemic, particularly attributed to COVID-19 vaccines. Influenza vaccines showed the highest association. In addition, vaccine-associated GBS had a higher association with older age groups. The TTO of vaccine-associated GBS was found to be an average of 5.5 days, occurring within one week. However, vigilant monitoring in high-risk groups identified from individual vaccines is crucial. In conclusion, these findings provide valuable insights into the global burden of vaccine-associated GBS, contributing to the development of safer vaccination protocols.

## Electronic supplementary material

Below is the link to the electronic supplementary material.

Supplementary Material 1

## Data Availability

The data are available upon request. Study protocol and statistical code: Available from DKY (yonkkang@gmail.com). Dataset: available from the Uppsala Monitoring Centre (WHO Collaborating Center) or WHO through a data use agreement.
