# Supplementary material for: Global burden of vaccine-associated Guillain-Barré syndrome over 170 countries from 1967 to 2023
Source: Sci Rep. 2024 Oct 19;14:24561. doi: 10.1038/s41598-024-74729-2 (PMC11490553; doi:10.1038/s41598-024-74729-2)
Supplement: Supplementary file 1 — Supplementary Material 1 [file 41598_2024_74729_MOESM1_ESM.docx]

| **Supplementary Material** |
| --- |

Research article

**Global burden of vaccine-associated Guillain-Barré syndrome over 170 countries from 1967 to 2023**

**Running title:** Vaccine-associated Guillain-Barré syndrome

Yi Deun Jeong,^1,2&^ Seoyoung Park,^2*^ Sooji Lee,^1,2&^ Woojin Jang,^1^ Jaeyu Park,^2,3^ Kyeongmin Lee,^2,3^ Jinseok Lee,^4^ Jiseung Kang,^5,6^ Raphael Udeh,^7^ Masoud Rahmati,^8,9,10^ Seung Geun Yeo,^11^ Lee Smith,^12*^ Hayeon Lee,^2,4^ Dong Keon Yon,^1,2,3,13*^

^&^ All authors contributed equally to this work.

**^*^Corresponding authors**

**Dong Keon Yon**, MD, PhD, FAAAAI, FACAAI

Center for Digital Health, Medical Science Research Institute, Kyung Hee University Medical Center, Kyung Hee University College of Medicine, 23 Kyungheedae-ro, Dongdaemun-gu, Seoul 02447, Republic of Korea

Tel: 82-2-958-8491, Fax: 82-2-958-8490

E-mail: [yonkkang@gmail.com](mailto:yonkkang@gmail.com)

**Seoyoung Park**, PhD

Center for Digital Health, Medical Science Research Institute, Kyung Hee University Medical Center, Kyung Hee University College of Medicine, 23 Kyungheedae-ro, Dongdaemun-gu, Seoul 02447, Republic of Korea

E-mail: [psy091222222@gmail.com](mailto:psy091222222@gmail.com)

**Lee Smith**, PhD

Centre for Health, Performance and Wellbeing, East Rd, Anglia Ruskin University, Cambridge CB1 1PT, UK

Email: Lee.Smith@aru.ac.uk

**Contents of Supplementary Materials**

| **Supplementary Material** | | Page |
| --- | --- | --- |
| **Table S1** | Matrix of vaccines associated with GBS | 3 |
| **Table S2** | Descriptions of vaccines associated with GBS including ATC codes | 4–6 |
| **Table S3** | Medical Dictionary for Regulatory Activities (MedDRA) preferred terms and classifications for GBS | 7 |
| **Table S4** | Medical Dictionary for Regulatory Activities (MedDRA) preferred terms for concurrent adverse reactions | 8–21 |
| **Table S5** | Multiple disproportionality analysis methods | 22 |

**Table S1.** Matrix of vaccines associated with GBS

|  | **Reports with a suspected GBS** | **Reports without a suspected GBS** |
| --- | --- | --- |
| **Reports with**  **suspected vaccines** | 15,377 | 7,995,225 |
| **All other reports** | 7,239 | 123,171,053 |

**Table S2.** Descriptions of vaccines associated with GBS including ATC codes

| **Drug type** | **ATC code** | **Drug description** |
| --- | --- | --- |
| **Vaccines** | **J07** |  |
| Rabies vaccines | J07BG |  |
| Yellow fever vaccines | J07BL |  |
| DTaP-IPV-Hib vaccines | J07AF, J07AG, J07AJ,  J07AM, J07BF, and J07CA |  |
| Meningococcal vaccines | J07AH |  |
| Pneumococcal vaccines | J07AL |  |
| Tuberculosis vaccines | J07AN |  |
| Typhoid vaccines vaccines | J07AP |  |
| Encephalitis vaccines | J07BA |  |
| Influenza vaccines | J07BB |  |
| Hepatitis A vaccines | J07BC | Aimmugen, Ambirix, Avaxim, Biovac a, Havrix, Epaxal, Hepatitis A, Hepatitis a vaccine, Hepatitis a vaccine inact, Hepatitis a vaccine; Hepatitis b vaccine, Twinrix, Vaqta |
| Hepatitis B vaccines | J07BC | Bimmugen, Engerix, Engerix b, Engerix-b, Elovac b, Euvax b, Fendrix, Gen h-b-vax, H-b-vax, H-b-vax ii, H-b-vax n, Hb, Hb vaccine, Hbvaxpro, Heberbiovac hb, Hepatitis B Vaccine recombinant, Hepatitis b vaccin, Hepatitis b vaccine, Hepatitis b vaccine r, Hepatitis b vaccine rHBsAg (yeast), Hepativax, Hepavax, Hepavax-gene, Heplisav b, Heptavax II, Heptavax-b, Recombivax hb, Recombivax-hb, Vaccin Genhevac B |
| MMR vaccines | J07BD, J07BE, and J07BJ |  |
| Rotavirus vaccines | J07BH |  |
| Varicella Zoster | J07BK |  |
| Papillomavirus vaccines | J07BM |  |
| COVID–19 mRNA vaccines | J07BN | Abdavomeran, BNT162a1, BNT162b1, BNT162b2, COVID-19 mRNA Vaccine BNT162b2, COVID-19 vaccine DNA, COVID-19 vaccine Moderna, COVID-19 vaccine mRNA, COVID-19 vaccine mRNA (BNT162b2), COVID-19 vaccine mRNA (mRNA 1273), COVID-19 vaccine mRNA 2v, COVID-19 vaccine mRNA S, Comirnaty, Comirnaty Original/Omicron BA.1, Comirnaty Original/Omicron BA.4-5, Comirnaty bivalente BA.1, Comirnaty bivalente BA.4/BA.5, Davesomeran;Elasomeran, Elasomeran, Elasomeran;Imelasomeran, Famtozinameran;Tozinameran, MRNA 1273, Moderna COVID-19 Vaccine, Bivalent (Original and Omicron BA.4/BA.5), Moderna COVID-19 vaccine, Pfizer BioNTech COVID-19 vaccine, Pfizer BioNTech COVID-19 vaccine, bivalent (original and omicron BA.4/5), Pidacmeran, Riltozinameran;Tozinameran, Spikevax, Spikevax bivalent Original / Omicron, Spikevax bivalent original/omicron ba.1, Spikevax bivalent original/omicron ba.4-5, Tozinameran, Vacuna COVID-19 Pfizer BioNTech, Vacuna Pfizer-BioNTech COVID-19, Zorecimeran |
| Ad5-vectored COVID–19 vaccines | J07BN | AZD 1222, AstraZeneca COVID-19 vaccine, AstraZeneca Korea Vaxzevria, COVID-19 vaccine AstraZeneca, COVID-19 vaccine Janssen, COVID-19 vaccine NRVV, COVID-19 vaccine NRVV Ad, COVID-19 vaccine NRVV Ad (ChAdOx1 nCoV-19), COVID-19 vaccine NRVV Ad26, COVID-19 vaccine NRVV Ad26 (Gam-Covid-Vac), COVID-19 vaccine NRVV Ad26 (Gam-Covid-Vac);COVID-19 vaccine NRVV Ad5 (Gam-Covid-Vac), COVID-19 vaccine NRVV Ad26 (JNJ 78436735), COVID-19 vaccine NRVV Ad5, COVID-19 vaccine NRVV Ad5 (Ad5-nCoV), COVID-19 vaccine NRVV Ad5 (Gam-Covid-Vac), COVID-19 vacuna AstraZeneca, ChAdOx1 nCoV-19, Convidecia, Covishield, Gam-COVID-Vac, Gam-COVID-Vac component 1, Gam-COVID-Vac component 2, JNJ 78436735, Janssen COVID-19 vaccine, Jcovden, Korea AstraZeneca COVID-19 vaccine, Recombinant COVID-19 Vaccine (Adenovirus Type 5 Vector), Sputnik V, Sputnik V component 1, Sputnik V component 2, Sputnik light, Vacina COVID-19 (recombinante), Vacuna COVID-19 AstraZeneca, Vacuna COVID-19 Janssen, Vaxzevria |
| Inactivated whole-virus  COVID–19 vaccines | J07BN | BBIBP-CorV, COVID-19 vaccine (Vero cell), inactivated, COVID-19 vaccine (inactivated, adjuvanted) valneva, COVID-19 vaccine (vero cell), inactivated, COVID-19 vaccine VLP, COVID-19 vaccine inact, COVID-19 vaccine inact (Vero), COVID-19 vaccine inact (Vero) 19nCoV-CDC-Tan-HB02, COVID-19 vaccine inact (Vero) CZ02, COVID-19 vaccine inact (Vero) HB02, COVID-19 vaccine inact (Vero) WIV04, COVID-19 vaccine inact w.virion (Erucov-vac), COVID-19 vaccine inact w.virion (QazCovid-in), COVID-19 vaccine inact w.virion (SARS-CoV-2/human/KAZ/KZ_Almaty/2020), COVID-19 vaccine inact w.virion (VLA 2001), CoronaVac, Covaxin, Covid-19 vaccine (vero cell), inactivated, Covilo, SARS-CoV-2 vaccine (vero cell), inactivada, SARS-CoV-2 vaccine (vero cell), inactivated, Turkovac, Vacina adsorvida COVID-19 (inativada), Vacuna SARS-CoV-2 (celulas vero) inactivada, Vacuna contra el SARS-COV-2 (Vero Cell), Inactivada, Vaksin COVID-19 bio farma |

Abbreviation: ATC, anatomical therapeutic chemical classification system; GBS, Guillain-Barré syndrome**.**

**Table S3.** Medical Dictionary for Regulatory Activities (MedDRA) preferred terms and classifications for GBS.

| **SOC** | **HLGT** | **HLT** | **PT** | **LLT** | **MedDRA code** |
| --- | --- | --- | --- | --- | --- |
| Nervous system disorders | Peripheral neuropathies | Acute polyneuropathies | Acute motor axonal neuropathy | Acute motor axonal neuropathy | 10076658 |
| Nervous system disorders | Peripheral neuropathies | Acute polyneuropathies | Acute motor-sensory axonal neuropathy | Acute motor-sensory axonal neuropathy | 10076657 |
| Nervous system disorders | Peripheral neuropathies | Acute polyneuropathies | Guillain-Barré syndrome | Acute infective polyneuritis | 10000813 |
| Nervous system disorders | Peripheral neuropathies | Acute polyneuropathies | Guillain-Barré syndrome | Acute inflammatory demyelinating polyradiculoneuropathy | 10067898 |
| Nervous system disorders | Peripheral neuropathies | Acute polyneuropathies | Guillain-Barré syndrome | Acute inflammatory demyelinating polyradiculopathy | 10067604 |
| Nervous system disorders | Peripheral neuropathies | Acute polyneuropathies | Guillain-Barré syndrome | AIDP | 10087910 |
| Nervous system disorders | Peripheral neuropathies | Acute polyneuropathies | Guillain-Barré syndrome | Guillain Barré syndrome | 10018766 |
| Nervous system disorders | Peripheral neuropathies | Acute polyneuropathies | Guillain-Barré syndrome | Guillain-Barré syndrome | 10018767 |
| Nervous system disorders | Peripheral neuropathies | Acute polyneuropathies | Guillain-Barré syndrome | Syndrome Guillain-Barré | 10042812 |
| Nervous system disorders | Peripheral neuropathies | Acute polyneuropathies | Miller Fisher syndrome | Fisher syndrome | 10076684 |
| Nervous system disorders | Peripheral neuropathies | Acute polyneuropathies | Miller Fisher syndrome | Miller Fisher syndrome | 10049567 |

Abbreviation: DRESS, drug reaction with eosinophilia and systemic symptoms; HLT, high-level term; HLGT, high-level group term; GBS, Guillain-Barré syndrome; LLT, lower-level terms; PT, preferred terms; SOC, system organ class.

**Table S4.** Medical Dictionary for Regulatory Activities (MedDRA) preferred terms for concurrent adverse reactions.

| **Concurrent adverse reaction subgroups** | **MedDRA preferred terms** |
| --- | --- |
| Coronary | Acute coronary syndrome, Acute myocardial infarction, Angina pectoris, Angina unstable, Arteriogram coronary abnormal, Arteriosclerosis coronary artery, Arteriospasm coronary, Arteritis coronary, Cardiac stress test abnormal, Catheterisation cardiac abnormal, Chest pain, Coronary arterial stent insertion, Coronary artery disease, Coronary artery dissection, Coronary artery occlusion, Coronary artery stenosis, Coronary artery thrombosis, Coronary vascular graft occlusion, ECG signs of myocardial ischaemia, Electrocardiogram ST segment abnormal, Electrocardiogram ST segment depression, Electrocardiogram ST segment elevation, Electrocardiogram ST segment normal, Electrocardiogram ST-T change, Electrocardiogram ST-T segment abnormal, Electrocardiogram ST-T segment elevation, Electrocardiogram T wave abnormal, Electrocardiogram T wave amplitude decreased, Electrocardiogram T wave inversion, Electrocardiogram T wave normal, Ischaemic cardiomyopathy, Myocardial infarction, Myocardial ischaemia, Myocardial necrosis, Myocardial necrosis marker, Myocardial necrosis marker increased, Myocardial reperfusion injury, Silent myocardial infarction, Troponin, Troponin I, Troponin I increased, Troponin increased, Troponin T, Troponin T increased, Vascular stent thrombosis |
| Arrhythmia | Arrhythmia, Atrial fibrillation, Atrial flutter, Atrioventricular block, Atrioventricular block complete, Atrioventricular block first degree, Atrioventricular block second degree, Bifascicular block, Bradycardia, Brugada syndrome, Bundle branch block, Bundle branch block left, Bundle branch block right, Cardiac ablation, Cardiac arrest, Cardiac electrophysiologic study abnormal, Cardiac pacemaker insertion, Cardiac telemetry abnormal, Cardio-respiratory arrest, Cardioversion, Conduction disorder, Defect conduction intraventricular, Electrocardiogram abnormal, Electrocardiogram ambulatory abnormal, Electrocardiogram P wave abnormal, Electrocardiogram P wave normal, Electrocardiogram PR interval, Electrocardiogram PR prolongation, Electrocardiogram PR shortened, Electrocardiogram Q wave abnormal, Electrocardiogram Q waves, Electrocardiogram QRS complex, Electrocardiogram QRS complex prolonged, Electrocardiogram QT interval, Electrocardiogram QT prolonged, Electrocardiogram QT shortened, Extrasystoles, Heart rate abnormal, Heart rate decreased, Heart rate increased, Heart rate irregular, Implantable defibrillator insertion, Lown-Ganong-Levine syndrome, Malaise, Palpitations, Presyncope, Pulseless electrical activity, Sinus arrhythmia, Sinus bradycardia, Sinus rhythm, Sinus tachycardia, Sudden cardiac death, Sudden death, Sudden infant death syndrome, Supraventricular extrasystoles, Supraventricular tachycardia, Syncope, Tachyarrhythmia, Tachycardia, Tachycardia paroxysmal, Torsade de pointes, Ventricular arrhythmia, Ventricular extrasystoles, Ventricular fibrillation, Ventricular tachycardia |
| Heart failure | Acute left ventricular failure, Acute pulmonary oedema, Atrioventricular septal defect, Brain natriuretic peptide abnormal, Brain natriuretic peptide increased, Cardiac dysfunction, Cardiac failure, Cardiac failure acute, Cardiac failure chronic, Cardiac failure congestive, Cardiac index decreased, Cardiac output decreased, Cardiac ventriculogram abnormal, Cardiogenic shock, Cardiomegaly, Cardiomyopathy, Cardiomyopathy acute, Cardiopulmonary failure, Cardiotoxicity, Circulatory collapse, Congestive cardiomyopathy, Diastolic dysfunction, Dyspnoea, Dyspnoea at rest, Dyspnoea exertional, Dyspnoea paroxysmal nocturnal, Echocardiogram abnormal, Ejection fraction abnormal, Ejection fraction decreased, Fluid overload, Intra-aortic balloon placement, Left ventricular dilatation, Left ventricular dysfunction, Left ventricular end-diastolic pressure increased, Left ventricular enlargement, Left ventricular failure, Low cardiac output syndrome, Myocardial depression, N-terminal prohormone brain natriuretic peptide increased, Oedema due to cardiac disease, Pulmonary arterial hypertension, Pulmonary arterial pressure increased, Pulmonary congestion, Pulmonary hypertension, Pulmonary oedema, Right ventricular dilatation, Right ventricular dysfunction, Right ventricular failure, Shock, Stress cardiomyopathy, Systolic dysfunction, Tachycardia induced cardiomyopathy, Ventricular assist device insertion, Ventricular dysfunction, Ventricular dyskinesia, Ventricular hypokinesia, Ventricular internal diameter abnormal, Ventricular septal defect, Orthopnoea, Dyspnoea |
| Other cardiac diseases | Acute myocarditis, Acute myocarditis in diseases classified elsewhere, Acute myocarditis, unspecified, Asymptomatic myocarditis, Biopsy pericardium, Cardiac tamponade, Focal myocarditis, Idiopathic myocarditis, Lymphoid interstitial myocarditis, Myocarditis, Myocarditis interstitial, Myocarditis NOS, Myocarditis, unspecified, Other acute myocarditis, Other and unspecified acute myocarditis, Pericardial disease, Pericardial drainage test normal, Pericardial effusion, Pericardial fibrosis, Pericardial haemorrhage, Pericardial rub, Pericarditis, Pericarditis constrictive, Pleuropericarditis, Polyserositis, Purulent pericarditis, Subclinical myocarditis, Toxic myocarditis, Viral pericarditis |
| Hyperthermia | Body temperature increased, Cytokine release syndrome, Cytokine storm, Delirium febrile, Feeling hot, Hot flush, Hyperpyrexia, Hyperthermia, Hyperthermia malignant, Inflammatory marker increased, Interleukin level increased, Neuroleptic malignant syndrome, Pyrexia, Serotonin syndrome, Systemic inflammatory response syndrome, Temperature regulation disorder |
| Eosinophilia | Drug reaction with eosinophilia and systemic symptoms, Eosinophil count, Eosinophil count increased, Eosinophil percentage, Eosinophil percentage increased, Eosinophilia, Eosinophilic myocarditis, Eosinophilic pneumonia, Hypereosinophilic syndrome, Pulmonary eosinophilia |
| Thrombocytopenia and leucopenia | Immune thrombocytopenic purpura, Platelet count decreased, Thrombocytopenia, Narcolepsy, Agranulocytosis, Aplasia, Bone marrow disorder, Bone marrow failure, Granulocytopenia, Leukopenia, Neutropenia, Neutrophil count decreased, Neutrophil percentage decreased, Pancytopenia, White blood cell count abnormal, White blood cell count decreased, White blood cell disorder |
| Pulmonary | Acute respiratory distress syndrome, Acute respiratory failure, Alveolitis, Asthma, Atelectasis, Autoimmune lung disease, Bradyphrenia, Bronchitis, Bronchospasm, Chronic obstructive pulmonary disease, Cor pulmonale, Cough, Crepitations, Diffuse alveolar damage, Emphysema, Haemorrhagic pneumonia, Hypoxia, Interstitial lung disease, Lung assist device therapy, Lung consolidation, Lung disorder, Lung infiltration, Obliterative bronchiolitis, Obstructive airways disorder, Pneumonia, Pneumonitis, Pulmonary alveolar haemorrhage, Pulmonary fibrosis, Pulmonary haemorrhage, Pulmonary toxicity, Traumatic lung injury, Hilar lymphadenopathy, Pleural effusion, Pleural fibrosis, Pleurisy, Pneumothorax |
| Infections | Abdominal infection, Abscess, Acinetobacter infection, Acute endocarditis, Adenovirus infection, Adenovirus test positive, Alpha haemolytic streptococcal infection, Alternaria infection, Appendicitis, Arthritis bacterial, Arthritis infective, Arthropod bite, Aspergillus infection, Atypical pneumonia, Avian influenza, Bacteraemia, Bacterial infection, Bacterial sepsis, Bacterial test positive, BK virus infection, Blood culture positive, Bronchiolitis, Bronchitis bacterial, Bronchitis viral, Candida infection, Candida test positive, Cardiac infection, Cardiac valve abscess, Clostridial infection, Clostridium difficile colitis, Clostridium test positive, Corneal infection, Corynebacterium test positive, Coxsackie virus test positive, Cryptococcosis, CSF red blood cell count positive, Culture positive, Culture throat positive, Cystitis, Cytomegalovirus infection, Cytomegalovirus test positive, Cytomegalovirus viraemia, Encephalitis viral, Endocarditis, Endocarditis staphylococcal, Endocarditis viral, Enterococcal bacteraemia, Enterococcal sepsis, Enterococcus test positive, Enterovirus infection, Enterovirus test positive, Epstein-Barr virus antibody positive, Epstein-Barr virus associated lymphoproliferative disorder, Epstein-Barr virus infection, Escherichia urinary tract infection, Febrile infection, Febrile neutropenia, Flavivirus test positive, Fungal infection, Gangrene, Gas gangrene, Gastroenteritis viral, Gastrointestinal infection, Graft infection, Gram stain positive, H1N1 influenza, Hepatitis B antibody positive, Hepatitis B core antibody positive, Hepatitis B core antigen, Hepatitis B surface antibody negative, Hepatitis B surface antibody positive, Hepatitis C, Hepatitis C antibody positive, Hepatitis viral, Herpes simplex, Herpes simplex test positive, Herpes zoster, Herpes zoster disseminated, Human polyomavirus infection, Human rhinovirus test positive, Infection, Infection in an immunocompromised host, Influenza, Influenza A virus test positive, Influenza B virus test positive, Influenza like illness, Influenza virus test positive, Injection site infection, Kidney infection, Laryngitis, Lower respiratory tract infection, Lower respiratory tract infection viral, Lyme disease, Lymphadenopathy, Lymphadenopathy mediastinal, Meningococcal infection, Meningococcal sepsis, Morbillivirus test positive, Mycoplasma infection, Mycoplasma test positive, Myocardiac abscess, Myocarditis infectious, Myocarditis post infection, Myocarditis septic, Nasopharyngitis, Neisseria infection, Neisseria test positive, Norovirus test positive, Oesophageal candidiasis, Osteoarthritis, Osteomyelitis, Otitis media viral, Pantoea agglomerans test positive, Parvovirus B19 test positive, Parvovirus infection, Pertussis, Pharyngitis, Pneumatosis intestinalis, Pneumocystis jirovecii infection, Pneumocystis jirovecii pneumonia, Pneumonia aspiration, Pneumonia bacterial, Pneumonia fungal, Pneumonia staphylococcal, Pneumonia viral, Polyomavirus-associated nephropathy, Post procedural pneumonia, Procalcitonin increased, Pseudomonas test positive, Pulmonary sepsis, Purpura fulminans, Respiratory syncytial virus infection, Respiratory tract infection, Respiratory tract infection viral, Respiratory viral panel, Rhinitis, Rhinovirus infection, Roseolovirus test positive, Sepsis, Septic embolus, Septic shock, Sinusitis, Sputum culture positive, Staphylococcal bacteraemia, Staphylococcal infection, Staphylococcal sepsis, Staphylococcus test positive, Stomatitis, Streptococcus test positive, Systemic mycosis, Tonsillitis streptococcal, Toxic shock syndrome, Trypanosomiasis, Upper respiratory tract infection, Urinary tract infection, Urinary tract infection enterococcal, Urosepsis, Vaccinia virus infection, Varicella zoster virus infection, Viral diarrhoea, Viral infection, Viral sepsis, Viral test positive, Viral upper respiratory tract infection, Wound infection, Mucormycosis |
| Abdominal (aseptic, non-hepatic) | Abdominal discomfort, Abdominal distension, Abdominal pain, Abdominal pain lower, Abdominal pain upper, Abdominal tenderness, Abnormal behaviour, Autoimmune colitis, Colitis, Colitis ulcerative, Crohn's disease, Diarrhoea, Diarrhoea haemorrhagic, Duodenitis, Enteritis, Enterocolitis, Frequent bowel movements, Gastric disorder, Gastric haemorrhage, Gastritis, Gastritis erosive, Gastroenteritis, Gastrointestinal disorder, Gastrointestinal haemorrhage, Gastrointestinal inflammation, Gastrointestinal motility disorder, Gastrointestinal necrosis, Gastrointestinal perforation, Gastrointestinal tube insertion, Gastrooesophageal reflux disease, Graft versus host disease in gastrointestinal tract, Ileus, Inflammatory bowel disease, Lipase increased, Megacolon, Melaena, Pancreatic failure, Pancreatitis, Pancreatitis acute, Perirectal abscess, Peritonitis, Retroperitoneal haematoma, Retroperitoneal haemorrhage, Serositis, Small intestinal haemorrhage, Small intestinal obstruction, Spleen disorder, Splenomegaly |
| Hepato-biliary | Acute hepatic failure, Alanine aminotransferase abnormal, Alanine aminotransferase increased, Ascites, Aspartate aminotransferase abnormal, Aspartate aminotransferase increased, Autoimmune hepatitis, Biliary anastomosis complication, Bilirubin conjugated increased, Bilirubin urine, Bilirubinuria, Blood alkaline phosphatase increased, Blood bilirubin abnormal, Blood bilirubin decreased, Blood bilirubin increased, Cholecystitis, Cholelithiasis, Cholestasis, Drug-induced liver injury, Gamma-glutamyltransferase abnormal, Gamma-glutamyltransferase increased, Hepatic atrophy, Hepatic cirrhosis, Hepatic congestion, Hepatic encephalopathy, Hepatic enzyme abnormal, Hepatic enzyme increased, Hepatic failure, Hepatic function abnormal, Hepatic haemorrhage, Hepatic necrosis, Hepatic pain, Hepatic steatosis, Hepatitis, Hepatitis acute, Hepatitis alcoholic, Hepatitis cholestatic, Hepatitis fulminant, Hepatocellular injury, Hepatomegaly, Hepatosplenomegaly, Hepatotoxicity, Hyperbilirubinaemia, Hyperplastic cholecystopathy, Hypertransaminasaemia, Immune-mediated hepatitis, Ischaemic hepatitis, Jaundice, Jaundice neonatal, Liver disorder, Liver function test abnormal, Liver function test increased, Liver injury, Nonalcoholic fatty liver disease, Portal tract inflammation, Transaminases increased |
| Endocrine | Adrenal cortex necrosis, Adrenal insufficiency, Adrenocortical insufficiency acute, Aldolase abnormal, Antidiuretic hormone abnormality, Autoimmune thyroiditis, Blood glucose abnormal, Blood glucose decreased, Blood glucose increased, Blood thyroid stimulating hormone decreased, Blood thyroid stimulating hormone increased, Diabetes insipidus, Diabetes mellitus, Diabetic ketoacidosis, Fulminant type 1 diabetes mellitus, Galactorrhoea, Glucose urine present, Glycosuria, Goitre, Gynaecomastia, Hyperglycaemia, Hyperlipidaemia, Hypermetabolism, Hyperprolactinaemia, Hypertension, Hyperthyroidism, Hypertriglyceridaemia, Hyperuricaemia, Hypophysitis, Hypopituitarism, Hypothyroidism, Ketoacidosis, Lymphocytic hypophysitis, Metabolic acidosis, Metabolic alkalosis, Metabolic disorder, Thyroid disorder, Thyroid mass, Thyroiditis, Thyroiditis acute, Thyroxine free decreased, Thyroxine free increased, Thyroxine increased, Tri-iodothyronine free increased, Type 1 diabetes mellitus, Type 2 diabetes mellitus, Hypoglycaemia |
| Muscular (Myositis & Myasthenia) | Biopsy muscle abnormal, Blood creatine phosphokinase abnormal, Blood creatine phosphokinase BB increased, Blood creatine phosphokinase increased, Blood creatine phosphokinase MB increased, Diaphragmatic paralysis, Electromyogram abnormal, Extraocular muscle paresis, Immune-mediated myositis, Muscle atrophy, Muscle disorder, Muscle injury, Muscle necrosis, Muscle rigidity, Muscle spasms, Muscle tightness, Muscle twitching, Muscular dystrophy, Muscular weakness, Musculoskeletal discomfort, Musculoskeletal disorder, Musculoskeletal pain, Musculoskeletal stiffness, Myalgia, Myopathy, Myositis, Necrotising myositis, Neuromyopathy, Orbital myositis, Polymyositis, Respiratory muscle weakness, Rhabdomyolysis, Use of accessory respiratory muscles, Diaphragmatic paralysis, Diplopia, Extraocular muscle paresis, Eyelid function disorder, Eyelid ptosis, Myasthenia gravis, Myasthenia gravis crisis, Myasthenic syndrome, Ophthalmoplegia, Respiratory depression, Respiratory fatigue, Respiratory muscle weakness, Use of accessory respiratory muscles |
| Neurologic | Agitation, Akathisia, Altered state of consciousness, Amnesia, Apathy, Aphasia, Areflexia, Asthenia, Ataxia, Brain injury, Brain oedema, Catatonia, Cerebral atrophy, Cerebral disorder, Cerebral haematoma, Cognitive disorder, Coma, Confusional state, Consciousness fluctuating, Craniocerebral injury, Delirium, Delusion, Dementia, Disorganised speech, Disorientation, Dizziness, Dizziness postural, Dysphagia, Dysphonia, Dysphoria, Dysplasia, Dysstasia, Dystonia, Encephalitis, Encephalomalacia, Encephalomyelitis, Encephalopathy, Epilepsy, Extrapyramidal disorder, Facial paralysis, Facial paresis, Fine motor skill dysfunction, Gait disturbance, Gait inability, Generalised tonic-clonic seizure, Guillain-Barre syndrome, Headache, Hemiparesis, Hemiplegia, Hiatus hernia, Hyperphagia, Hypertonia, Hypoaesthesia, Hypoaesthesia oral, Hypokinesia, Hypophagia, Hyporesponsive to stimuli, Hypotonia, Hypotonic-hyporesponsive episode, Idiopathic intracranial hypertension, IIIrd nerve disorder, Incoherent, Intraventricular haemorrhage, Irritability, Laurence-Moon-Bardet-Biedl syndrome, Lethargy, Logorrhoea, Loss of consciousness, Lumbar puncture, Meningitis, Mental disorder, Mental disorder due to a general medical condition, Mental impairment, Mental status changes, Migraine, Miller Fisher syndrome, Mobility decreased, Motor dysfunction, Movement disorder, Myelitis, Nausea, Nervous system disorder, Nervousness, Neuralgia, Neuritis, Neuropathy peripheral, Neurotoxicity, Optic neuritis, Paraesthesia, Paralysis, Paresis, Parkinsonism, Parkinson's disease , Parosmia, Peripheral sensory neuropathy, Persecutory delusion, Personality disorder, Physical disability, Poliomyelitis, Postictal state, Pseudodementia, Quadriplegia, Seizure, Spinal compression fracture, Status epilepticus, Subdural haematoma, Tonic clonic movements, Toxic encephalopathy, Toxic neuropathy, Tremor, Trismus, Vertigo, Vestibular disorder, VIth nerve paralysis |
| Psychiatric | Abnormal dreams, Aggression, Anxiety, Anxiety disorder, Behaviour disorder, Bipolar disorder, Completed suicide, Crying, Decreased appetite, Depressed level of consciousness, Depressed mood, Depression, Depression suicidal, Feeding disorder, Gambling disorder, Generalised anxiety disorder, Hallucination, Hallucination, audutory, Hallucination, olfactory, Hallucinations, Homicidal ideation, Hypersomnia, Insomnia, Intellectual disability, Intentional self-injury, Language disorder, Major depression, Mania, Memory impairment, Middle insomnia, Mutism, Obsessive-compulsive symptom, Paranoia, Post-traumatic stress disorder, Psychiatric decompensation, Psychiatric symptom, Psychotic behaviour, Psychotic disorder, Psychotic symptom, Rebound psychosis, Schizoaffective disorder, Schizophrenia, Screaming, Selective eating disorder, Sense of oppression, Sexually inappropriate behaviour, Sleep disorder, Sluggishness, Somatic delusion, Somnolence, Speech disorder, Substance-induced psychotic disorder, Suicidal ideation, Tearfulness |
| Osteoarticular & rheumatologic | Anti-neutrophil cytoplasmic antibody positive vasculitis, Antinuclear antibody positive, Arthralgia, Arthritis, Arthropathy, Bone disorder, Bone pain, Central nervous system vasculitis, Connective tissue disorder, Costochondritis, Double stranded DNA antibody, Dysarthria, Epicondylitis, Foot fracture, Gout, Gouty arthritis, Hip fracture, Hypersensitivity vasculitis, Infectious mononucleosis, Infective exacerbation of chronic obstructive airways disease, Joint noise, Joint stiffness, Joint swelling, Lupus nephritis, Lupus-like syndrome, Osteoporotic fracture, Periarthritis, Pulmonary vasculitis, Rheumatoid arthritis, Rheumatoid factor increased, Sarcoidosis, Sjogren's syndrome , Soft tissue disorder, Systemic lupus erythematosus, Systemic scleroderma, Takayasu's arteritis , Tibia fracture, Vasculitis, Antiphospholipid syndrome |
| Dermatologic | Acne, Acute febrile neutrophilic dermatosis, Acute generalised exanthematous pustulosis, Alopecia, Alopecia totalis, Aphthous ulcer, Burning sensation, Cellulitis, Dermatitis, Dermatitis acneiform, Dermatitis bullous, Dermatitis contact, Dermatitis exfoliative, Dermatitis exfoliative generalised, Eczema, Erythema, Erythema multiforme, Erythema nodosum, Exfoliative rash, Folliculitis, Graft versus host disease in skin, Necrotising fasciitis, Nodular rash, Pain of skin, Papule, Pemphigus, Petechiae, Pruritus, Psoriasis, Rash, Rash erythematous, Rash macular, Rash maculo-papular, Rash morbilliform, Rash papular, Rash pruritic, Rash pustular, Skin candida, Skin discolouration, Skin disorder, Skin exfoliation, Skin fissures, Skin laceration, Skin lesion, Skin mass, Skin necrosis, Skin odour abnormal, Skin ulcer, Skin warm, Stevens-Johnson syndrome, Tardive dyskinesia, Toxic epidermal necrolysis, Urticaria, Vaccination site cellulitis, Vaccination site erythema, Vaccination site eschar, Vaccination site reaction, Vaccination site scab, Vaccination site swelling, Vaccination site vesicles |
| Ophthalmology | Amblyopia, Blindness, Blindness transient, Cataract, Conjunctivitis, Dry eye, Eye disorder, Iridocyclitis, Miosis, Mydriasis, Ocular hyperaemia, Periorbital oedema, Photophobia, Retinal pigment epitheliopathy, Retinal pigmentation, Retinopathy, Tunnel vision, Uveitis, Vision blurred, Visual field defect, Visual impairment |

**Table S5.** Multiple disproportionality analysis methods.

| **Measure of association** | **Definition** | **Criteria for signal detection** |
| --- | --- | --- |
| Information component (IC) | $\log_{2} \frac{N_{observed}+ 0.5}{N_{expected}+ 0.5}$ | ≥0 |
| Reporting odds ratio (ROR) | $\frac{A/B}{C/D}$ | ≥1 |

The IC was computed assuming a Bayesian analysis for case-non-case analysis.^21^ It serves as an indicator value for disproportionate reporting, comparing observed and expected ADR associations to identify the drug-ADR signals with a probability difference from the background data. ROR was calculated using the following formula: ROR = (a/b) / (c/d), where "a" represents the number of reports for a certain adverse drug reaction, "b" is the number of reports for all other ADRs with a specific drug, "c" is the number of all reports for certain ADRs not related to a specific drug, and "d" is the number of all reports not related to both specific ADRs and drugs. An IC_025,_ the value representing the lower end of the 95% confidence interval of the IC, greater than 0.00 and ROR>1.00 indicate statistical significance. It means that cases are more reported with the drug of interest than with other drugs, same as the greater the disproportionality. The IC_025_ and ROR, being statistical estimates, should always be presented and interpreted with a 95% confidence interval (95% CI).
